# Psychological Lockdown Experiences: Downtime or an Unexpected Time for Being?

**DOI:** 10.3389/fpsyg.2021.577089

**Published:** 2021-04-08

**Authors:** Fortuna Procentese, Ciro Esposito, Florencia Gonzalez Leone, Barbara Agueli, Caterina Arcidiacono, Maria Francesca Freda, Immacolata Di Napoli

**Affiliations:** Department of Humanities, University of Naples Federico II, Naples, Italy

**Keywords:** time, lockdown, storytelling, well-being, young adults, confined in the present, confined in the past, striving toward one’s goals

## Abstract

The spread of COVID-19 in Italy resulted in the implementation of a lockdown that obligated the first time the general populace to remain at home for approximately two months. This lockdown interrupted citizens’ professional and educational activities, in addition to closing shops, offices and educational institutions. The resulting changes in people’s daily routines and activities induced unexpected changes in their thoughts, feelings and attitudes, in addition to altering their life perceptions. Consequently, the present study explores how young adults perceived their lives under lockdown during the final week of March 2020, when the reported number of daily coronavirus infections reached its peak in Italy. The research was carried out among 293 university students (234 women and 59 men) with an average age of 20.85 years old (SD = 3.23). The researchers asked participants to describe the emotions, thoughts and experiences that characterized their time under lockdown. The study analyzed specific narratives related to time and space using grounded theory methodology, which was applied using Atlas 8 software, leading to the creation of 68 codes. The study organized these codes into three specific categories: confined in the present, confined in the past, and striving toward one’s goals. Finally, the researchers also created a core-category labeled “continuity of being.” The results showed that the closure of open spaces caused a division in participants’ perceptions of time continuity, with many viewing themselves as feeling fragmented and as living the present in a static and fixed way. Additionally, participants also saw the present as being discontinuous from the past, while, simultaneously, projecting toward the future and the changes it might bring. Finally, this study examined further implications surrounding individual projecting among young people in greater depth.

## Introduction

The first COVID-19 emergency lockdown in Italy was in place from March 9 to May 4, 2020. During this period, the daily lives of Italians were turned completely upside down, as various government decrees restricted routine educational, professional and social activities. Recent studies on the COVID-19 emergency have highlighted that forced isolation has had a great impact on people’s psychological condition (Brooks et al., 2020; Duan and Zhu, 2020; Pancani et al., 2020).

In particular, many studies have focused on the lockdown’s impacts on children and adolescents (Orgilés et al., 2020), women vulnerable to domestic violence (Troisi, 2020) and students distress (Cao et al., 2020; Zurlo et al., 2020). Particularly, Di Napoli et al. (2021) and Migliorini et al. (2021) described that students’ individual feelings were characterized by fear, distress, sadness, rage, and loneliness, which are detailed in conjunction with coping strategies and resilience, as well as interpersonal relations at the familial and institutional levels (Marzana et al., 2021).

Further variables associated with higher levels of depression, anxiety and stress were female gender, negative affect and detachment (Mazza et al., 2020) but people with more resilient coping strategies were more likely to experience fewer depressive symptoms (Roma et al., 2020a).

Moreover, psychological factors, such as self-efficacy, risk perception and civic engagement, had a central role in the adherence to the measures adopted by the institution to contain the spread of the virus (Roma et al., 2020b).

New individual and social insights about “we-ness” awareness arise (Walker et al., 2020), strengthening solidarity, connectedness and shared experiences of “we-ness,” and improving individual and community well-being (Procentese et al., 2020; Di Napoli et al., 2021).

However, no known studies have investigated how the pandemic has affected people’s subjective perceptions of time.

As observed in the biographies of those who have lived through experiences such as spending time in prison, forced confinement influence one’s perceptions of time. For example, in his biography, Nelson Mandela (1994) sheds light on his many years in prison, describing how, even in the worst detention, he never gave up on his dreams and hopes for freedom, while constantly developing plans for future action. Indeed, many psychological researchers have investigated individuals’ attitudes toward temporal perspectives. For example, Stolarski et al. (2018) examined the long history of time and its deep roots in philosophy and physics. Additionally, Zimbardo and Boyd (2015) defined time perspectives as individual subjective experiences through which each person refers to the psychological concepts of the past, present and future. Zimbardo and Boyd (2015) specifically elaborated five individual attitudes toward time, encompassing future, past-negative, past-positive, present-hedonistic, and present-fatalistic, which are correlated with differing levels of well-being. In particular, individuals possessing past-positive and present-hedonistic time perspectives are thought to have higher levels of well-being, while future time perspectives are associated with optimism, hope and an internal locus of control.

More recently, Prilleltensky et al. (2015) proposed a concept of well-being specifically measured using a temporal perspective, assuming that individual perceptions of well-being cannot ignore experiences from the past, those perceived in the present and those imagined in the future (Di Martino et al., 2018; Di Napoli et al., forthcoming). Moreover, the seminal contribution of Pichon-Rivière on time and space in everyday life pointed out that everything that happens in daily life takes place in a given space and at a given time. Furthermore, as expressed by Quiroga and Racedo, these routine events possess a “rhythm” that is shaped by “the complex social relationships that govern the life of human beings in a certain historical era” (2012, p.18). In addition, external events alter how time and space frame individuals’ daily lives and undermine our concrete conditions of existence. However, “only when everyday life hurts us, when there is no more pleasure or when we experience a crisis, [do] we start to think about it” (Quiroga and Racedo, 2012, p.18).

In principle, from an ecological perspective, individual dimensions and social events influence behaviors, as every external change modifies people’s reference frameworks, and, in turn, these changes alter one’s internal world and their world perceptions (Lewin, 1943). Earlier research has considered how particular sudden and unexpected events, such as serious illnesses, natural disasters, terrorist attacks, wars, geographical relocation or temporary time constraints influence time perspectives (e.g., Fung et al., 1999; Fung and Carstensen, 2004). Indeed, some studies have found that well-being is also strictly related to subjective experiences of these events (Petrillo et al., 2014). From a phenomenological point of view, illness, as with any other critical event, causes a break in one’s sense of continuity, in addition to dramatic experiences of discontinuity. This perspective underlines a crisis of the subject’s sense-making systems and the necessity to pursue meaning, in order to interpret ongoing experiences. Moreover, critical events and illness represent elements of discontinuity that saturate the present and the entirety of sense-making processes. During the perturbation phase, time is organized through extremely subjective modalities, appearing to be confusing and inconsistent at first glance. However, a deeper analysis regarding the effects of illness on time perceptions, such as the dyscrasia of the temporal order, indicates that time perceptions are the result of the ongoing sense-making processes concerning what is happening at that moment. In this regard, how illness affects time perceptions involves not only meaning construction in response to the rupture of canonical states, but also a separate process of crisis subjectification. In other words, illness-influenced time perceptions result in significant sense-making processes aimed at the construction of these perceptions (Freda et al., 2015, p. 209). As an example, people with chronic diseases when they suffer the unpredictable vulnerability of their illness are considering their selves under the Damocles Syndrome, in allusion to the Greek mythological tale about an imminent and ever-present peril (Gonzalez Leone et al., 2019). Consequently, i.e., oncology patients are worried and anxious about eventual cancer recurrence comprising a second pathology related to impending distress or possible danger that may materialize at an unknown time in the future.

Concerning natural disaster-related emergencies, such as floods, earthquakes and pandemics, common sense reflections emphasize their catastrophic aspects. However, these circumstances take also on unexpected dimensions, such as changes in relational and time perspectives. Consequently, significant external events cause time and space new perceptions, reconstructing individuals’ inner worlds and their connections with their environmental frameworks.

In addition, according to the community psychology perspective utilized by the present study, space and time are both framed also by environmental, social and relational circumstances (Orford, 2008).

### Future Time Perceptions

Through-out life, people must adapt to circumstances that require the integration of new life events and changes (Kralik et al., 2006). Subjectively perceiving future time is essential because it is associated with one’s choice of goals, objectives and preferences (Carstensen et al., 1999) at different ages (Donizzetti, 2019).

Life transition events lead to changes in time perceptions (Wittmann and Lehnhoff, 2005), especially those regarding the future (Carstensen et al., 1999).

In particular, life transitions are moments where time continuity is broken because of changes in temporal perspectives that result in a division between past experiences and future plans.

This implies that young adults seek information and knowledge regarding social goals, in order to maximize their future opportunities. Young adulthood is in fact characterized by new identity exploration and development and is often coupled with significant life changes, such as entering the labor market, achieving financial independence and establishing long-term intimate relationships (Arnett, 2000; Crocetti et al., 2012). This transition to adulthood is also imbued with optimism, given young adults’ enhanced capacity to explore the world and envisage new life trajectories (Larson et al., 2002; Arnett, 2007). On the contrary, time perceptions among the elderly are more limited. Elderly individuals have limited time perceptions and are more motivated to pursue emotion-focused goals (Carstensen et al., 2006; Charles and Carstensen, 2010).

However, in the words of Rosa (2003), Italian youth’s existence has also become de-temporalized, as “Life is no longer planned along a line that stretches from the past into the future; instead, decisions are taken from ‘time to time’ according to situational and contextual needs and desires” (p.19).

Therefore, listening to the voices of Italian youth during the peak of the unexpected COVID-19 pandemic let researchers deepen their understanding of how time perceptions could affect young people confined at home, who generally already perceived the future in uncertain, accelerated and fragmented terms (Leccardi, 2005).

To better understand time perceptions and representations during the lockdown, we conducted a research with Italian university students to document their personal experiences of this unexpected and unforeseen event.

From this perspective, understanding young adults’ time perceptions while being confined at home due to the COVID-19 pandemic would provide valuable contributions to the existing literature.

In this light, the present article seeks to expand upon the existing research, by elaborating upon the effects of the COVID-19 lockdown on young Italian students’ psychological perceptions of time.

This study analyses students’ reports regarding their personal lives during home confinement. Generally, time is “taken into account in terms of life events and life experiences and, therefore, refers to a life-course perspective that is often processed in retrospect” (Kieslinger et al., 2020, p.1). In this case, however, our participants presented their present perceptions of their lives during forced confinement at home. Therefore, their perspectives represented a view of life in confinement and their texts described the meanings they attributed to time perceptions while confined at home.

This study sought to explore the effects of the sudden life changes resulting from the COVID-19 lockdown, in addition to examining their implications and impacts on participants’ daily lives, emotions and thoughts. Specifically, this study further investigated the thoughts and emotions related to time perceptions in a confined space, seeking to expand upon the existing knowledge regarding any related impacts on well-being or distress. The researchers collected data in Italy during the last week of March 2020, when the Italian government decreed a total lockdown as measure to contain the spread of COVID-19.

## Materials and Methods

### Participants

The research participants were composed of psychology students from the University of Naples Federico II. Recruitment occurred during online lessons, via invitations from teachers inviting students to share their thoughts about the lockdown through the digital platform, SurveyMonkey. The study involved a total of 293 students, including 234 females and 59 males, between 19 and 29 years old, with an average of 20.85 years (SD = 3.22). The strong gender disparity is representative of the general population of psychology students in Italy, in which women comprise 77.6% of students (Centro Studio Investimenti Sociali, 2019). Table 1 displays participants’ sociodemographic characteristics.

**TABLE 1:** Participant characteristics.

*Age*	*M* = 20.85	*SD* = 3.22
	*%*	*N*
**Sex**		
Male	20.1	59
Female	79.9	234
**Housing Situation**		
Living with one or both parents	90.8	266
Living alone	0.7	2
Living with a partner	1.0	3
Living with one or more roommates	3.1	9
Living with other family members	4.4	13
**Total**	100	293

### Methods and Procedures

This study used a storytelling approach, collecting individual and relational experiences with the aim of creating shared awareness. Therefore, storytelling was much more than a device to deepen individual biographical paths, as it also served as a tool for shared support, common identification and social change. The researchers collected students’ texts using the online platform SurveyMonkey as a teaching tool to support an online undergraduate community psychology course. The study asked participants to report their thoughts and emotions related to their lockdown experiences, as well as any other actions or events that they wished to share, limiting their written contributions to 10,000 characters or less. In the context of this study, it is pertinent to highlight the importance of using qualitative methods to detect shared meaning in individual stories using specific, defined procedures (Rappaport, 1995), which allow for meanings attributed to events to be detected, deepening the general knowledge regarding the subject and uncovering new perspectives and ideas (Salvatore et al., 2018).

### Data Analysis

We conducted the textual analysis using grounded theory methodology (GTM), which develops theoretical frameworks through the close examination of participants’ narratives. It is a bottom up, qualitative approach, in which findings emerge from the data, in accordance with Glaser’s (1992) principles. The use of GTM found receptive audiences among psychology and community-based researchers (Stewart, 2000; Rasmussen et al., 2016). The research team developed its coding activities using a bottom-up approach that was not based on *a priori* categories. This entails an “iterative process proceeding from substantive to theoretical coding. Grounded theorists proceed from the relationships between indicators in the data to the relation of these indicators to larger categories… The distinction between substantive and theoretical codes is the difference between the content observed in the data and what researchers theorize about that content” (Rasmussen et al., 2016, p. 25).

Grounded Theory is a methodology applied to qualitative research that involves the construction of hypotheses and theories through the collecting and analysis of data and for this reason it is used in all those researches in which there is no well-defined starting hypothesis.

Indeed, it was already used in other research that have deepened the psychological impact of the COVID-19 emergency (Di Napoli et al., 2021; Marzana et al., 2021; Migliorini et al., 2021).

The researchers began by open coding the texts and then grouping codes into larger categories in order to more fully understand the texts’ proposed meanings. Namely, this study structured data analysis in 5 phases: (a) familiarization with the data, (b) initial code generation, (c) grouping the codes and their subsequent review, (d) defining and labeling the codes, and (e) creating code macro-categories and describing any relationships among them. Throughout all five steps, the research team interacted and discussed meanings using their reflective competences, agreeing up content definitions through these reciprocal and collective thought interactions.

For the data analysis Atlas 8 software (Muhr, 2017) was used.

## Results

The analysis of the textual material resulted in 68 codes. The researchers subsequently organized these codes into 10 groups, which they later divided in three macro-categories (see Table 2). The results section elaborates on these macro-categories, code groups and some particularly explanatory quotations in greater detail.

**TABLE 2:** Coding process.

Code	Codes group	Macro-categories
*A feeling of emptiness*; *a sense of immobility*; *a sense of strangeness due to absence of contact*; *a suspension of daily life*; *deserted cities*; *endless days; every day is the same*; *frozen time*; *normality on hold*.	**Frozen time**	***CONFINED IN THE PRESENT***
*Absence of an end*; *concern about not returning home*; *feeling caged in*; *impossibility of returning to one’s home country*; *quarantined country*; *the absence of an escape route*; *travel interruptions*.	**Trapped in the present**	
*A surreal situation*; *chaos*; *feeling destabilized*; *feeling overwhelmed by the situation*; *loss of a life balance*; *sense of bewilderment.*	**Lost in time and space**	
*Birthday in quarantine*; *change of attitude toward others*; *importance of the present*; *new form of social relations*; *new interests*; *new situation*; *sudden lifestyle changes*.	**New life routines**	
*Inadequacy of past behaviors*; *past-present comparison*; *regret the past*; *revaluation of social relationships*; *revaluation of what you have*.	**Revalue the past**	***CONFINED IN THE PAST***
*A lack of freedom*; *a loss of privacy*; *a need for multiple spaces*; *a need to return to one’s daily life*; *a sense of invasion*; *forced cohabitation*; *forced to stay at home*; *I can’t wait to relive normalcy*; *importance of normality*; *imposed free time*.	**Return to normality**	
*Carpe diem*; *life is unpredictable*; *live life to the fullest*; *live now and not tomorrow*; *revaluation of life*; *revaluation of time*; *time is precious*.	**There is no time to waste**	***STRIVING TOWARD ONE’S GOALS***
*Distancing oneself from today and embracing tomorrow*; *future prospects*; *interest in future implications*; *tolerating uncertainty*; *uncertainty about the future*.	**Living in the present, but in anticipation of the future**	
*A unique experience*; *being alone with yourself*; *opportunity for change*; *opportunity to reflect*; *situation that marks you*; *stop and think*.	**Turning point**	
*Achieving a new life balance*; *evolution of oneself*; *greater awareness*; *personal growth*; *rediscover oneself*; *revaluation of oneself*.	**Self-evolution**	

### Confined in the Present

This macro-category is comprised of code groups that refer to participants’ perceptions of the present time. It is indicative of how young people involved in the study dealt with the passage of time during the lockdown at home in a confined space. In fact, the study revealed four distinct code groups that were “*confined in the present*,” and which reported different ways of relating to daily life during the COVID-19 state of emergency.

One identified code group encompassed participants whose time perceptions could be characterized as “*frozen time*,” in the sense that they perceived the lockdown as impeding their routine professional, educational and leisure activities. Codes that characterized this category highlighted a condition of immobility, as well as one of estrangement and detachment from people and daily life routines and habits, as illustrated by the excerpt below (*normality on hold*, *a suspension of daily life activities*, *every day is the same*), as illustrated by one participant’s thoughts “*We were forced to stop, to change our habits, to deal with a reality that no one had ever imagined possible.*”

These participants perceived time as frozen (*a sense of immobility*) and lacking direction in the present, as if they were lost in a desert without anything around, bereft of life, movement and relationships. Moreover, participants experiencing “*frozen time*” indicated that they lacked any interest in partaking in potentially different experiences, as they possessed little notion of time and also viewed outdoor spaces as empty and devoid of life (*deserted cities*), as illustrated by one participant’s thoughts “*Life slowed down and then stopped. My village became silent in an instant. We were so rowdy, sentimental, ‘physical,’ passionate and friendly with everyone. This all stopped. Now, we no longer meet. We no longer touch each other.*”

A second identified code group regarding how participants experienced everyday life during the lockdown comprised those who were “*trapped in the present.*” This code group refers to the difficulties that some young people experienced during the state of emergency, characterized by social isolation and forced confinement. In this context, many young adults who were “trapped in the present” perceived themselves as being locked up and unable to move. In fact, participants often reported a perception of being trapped, which they frequently characterized as unescapable or lacking a defined exit (*feeling caged in*, *the absence of an escape route*, as illustrated by one participant’s thoughts “*But, at the same time, unfortunately, it has become a ‘cage’ that keeps me locked in these walls and does not allow me to breathe.*”

In addition, many participants who were “*trapped in the present*” emphasized a sense of unease due to their inability to leave their respective homes and/or countries (*quarantined country, travel interruptions*), or, conversely, to return to their home countries (*impossibility of returning to one’s home country*), as illustrated by one participant’s thoughts “*The day the red zone was established in all of Italy, I was with my boyfriend in Holland. We were supposed to have stayed there for a week on vacation. As a result, we were constantly worrying about not being able to go home in the following days.*”

The third code group was comprised of participants whose perceptions could be described as “*lost in time and space*,” alluding to the negative experiences generated by the expansion of the pandemic. Many participants reported having undergone moments where they felt lost and thought they would not be able to cope with the emotional burdens resulting from the pandemic (*a surreal situation*, *a sense of bewilderment*, *feeling overwhelmed by the situation*), as illustrated by one participant’s thoughts “*We are living in a moment that could be defined as a cyclone of emotions. We feel completely overwhelmed.*”

In fact, this dramatic, unprecedented situation has led many participants to experience confusion and to lose sight of their past reference points (*chaos*, *feeling destabilized*) “*This situation shocked me because it forced me to reorganize my daily life. It pushed me away from the university life routines that I had previously created.*”

Finally, the fourth code group consisted of texts who viewed the lockdown as a chance to adopt “*new life routines*,” highlighting a way of dealing with the present by emphasizing the positive aspects of confinement at home. These codes refer to participants who demonstrated proactive attitudes and an ability to dedicate themselves to exploring new interests in their free time, in addition to those who embraced this new lifestyle (*new interests*, *sudden lifestyle changes*) “*Then, I rediscovered my interests, which I had not cultivated for a long time. I started reading, writing, cooking, playing and drawing. And this is another thing that I will carry with me, and that I hope to continue doing, even when this situation is over.*”

In addition, participants whose time perceptions were shaped by their “*new life routines*” emphasized their ability to maintain and manage interpersonal relationships and active ties with family and friends, despite spatial distances, thanks to the use of social networks. Among this code group, the inability to see others in-person both enhanced and deepened their social ties in certain ways (*new forms of social relations*) “*We try to stay close, thanks to video calls, even if we are physically far apart.*”

Finally, participants who embraced “*new life routines*” under lockdown underlined both their ability and desire to celebrate life events, albeit in alternative ways. They celebrated parties with relatives and friends all the same, through group calls on social networks (*birthdays in quarantine*) “*We still celebrated my friends’ birthdays, despite the fact that they occurred during the weeks under lockdown. It was strange, but still beautiful!*”

### Confined in the Past

This macro-category contains all code groups that refer to participants who indicated tendencies to escape from the present by taking refuge in the past. Many young people reported a desire to return to the past, prior to the pandemic and the lockdown. This macro-category is comprised of two specific code groups, whose participants both evoked memories of their earlier lives and/or desired past actions that they did not take.

The first code group consisted of participants who sought to “*revalue the past*,” referring to their desire to change certain aspects of their past, resume suspended activities or start new activities that were made impractical by the lockdown. For example, many participants reported revaluing their past, with a particular focus on their interpersonal relationships (*revaluation of social relationships*) and on aspects of their lives that they considered to be valuable, but not normally not appreciated and often taken for granted (*revaluation of what you have*) “*In this dark moment, I learned how important is to appreciate what we have, without taking things for granted, how fundamental it is to love normality every day, to dedicate more time to the people we love and to stop every now and again and reflect. It allowed me to understand the irreplaceability of physical contact and embracing one another, as well as the need for reciprocity.*”

In some cases, participants also expressed awareness in conjunction with certain feelings of guilt related to their failures to put prevention and common sense’ behaviors into practice earlier in the pandemic, which could have minimized the spread of COVID-19 (*inadequacies of past behaviors*) “*The situation we are experiencing now is a reflection of the initial attitudes that everyone had toward this virus. We have been too irresponsible.*”

The second code group consisted of participants whose time perceptions focused on a “*return to normality*,” in reference to their desires to restore pre-pandemic norms as soon as possible and to resume their pre-pandemic daily lives, composed of actions, spaces and consolidated times. In fact, students often referred to the desire to regain their freedom, understood as the need to regain control of their daily time management. In particular, they strove to restore the division between moments dedicated to productive activities (work or study) and leisure time, which often lacked a clear partition during the lockdown (*a lack of freedom*, *a need to return to one’s daily life*; *imposed free time*) “*It is not free time that is at my disposal now, since it is not time that I voluntarily chose to dedicate to myself. It is time that has imposed itself on me.*”

Participants’ references to their living spaces comprise another important aspect of this code group. Some participants described their homes, which typically embodied feelings of safety and protection, as places of confinement similar to a prison, given the limits they imposed upon participants’ freedoms. The lockdown also forced family members to share domestic spaces for much longer than in the pre-pandemic past, leading many young people to feel that their physical and mental spaces were being invaded (*a need for multiple spaces*, *forced cohabitation*, *a sense of invasion*) “*I have to adapt slowly, even if it is very difficult at 20 years old to live with 6 people in a 4-room house and to share a room with two younger sisters.*”

### Striving Toward One’s Goals

The final macro-category includes four code groups that focus on a sort of “time travel” on the part of the study participants. This journey led participants to explore new time perceptions that they had never experienced before, in addition to well-known domestic spaces that were viewed in a different light. This journey also led participants to reassess their lives and enhanced their desire to live without wasting a moment, leading them to evolve, to seek new experiences and to imagine a new future. The first code group can be characterized by a philosophy based upon the belief that, “*there is no time to waste.*” In fact, this idea encapsulates the reflections of many participants regarding life’s value and the fact that unpredictable events, such as the COVID-19 pandemic, can occur and lead to limitations and suffering. The thought of the possibility that unpredictable events may occur led many participants to reconsider the importance of living life to the fullest, to avoid postponing important decisions and actions and to experience the present by appreciating small moments of happiness in life (*carpe diem*, *time is precious*, *life is unpredictable*, *live life to the fullest*) “*This experience is teaching me that life must always be enjoyed, every day*”; “*I have never realized the importance of time in my life. We young people lose sight of time’s importance every day. We do not realize how much it really matters, and the fact that it would be beneficial to stop for a moment to reflect upon this.*”

The second code group consisted of participants who were “*living in the present, but in anticipation of the future.*” These participants’ time perceptions were focused on the ways in which they lived through the lockdown, possessing an evasive attitude toward the present, which was viewed as a period that they must get through in order to have a brighter future. In fact, the responses of participants “*living in the present, but in anticipation of the future*” often referred to a time in the future when it will be possible to resume physical contact with others and when the pandemic will be just a bad memory (*tolerating uncertainty*, *distancing oneself from today and embracing tomorrow*, *future prospects*) “*We must endure this moment. It is difficult, but we know it will pass. It must pass! We have to think about the future, which will surely be better. Everything will be fine!*”

The third future-focused code group viewed the pandemic as a sort of “*turning point.*” Their reflections characterized this tragic event as an evolutionary crisis that marked a turning point in the lives of individuals and the community. These participants saw the obligation to stay at home as an opportunity to stop and think about their own lives and the directions that they had taken thus far, as well as the course of their future lives following their lockdown experiences (*stop and think*, *an opportunity to reflect*) “*This is a time that allows for introspection, allows you to talk to yourself and to listen to your ego. It is a time that allows us to discover, to rest, to recharge, to show our creativity and to escape the fast-paced society to which we have become accustomed. It is a time to understand that everything is fine and beautiful and that we have to listen to the silence, a silence that submerges us and which almost seems unnatural and stunning in its intensity.*”

The uniqueness of the lockdown experience has led participants to believe that their future attitudes and behaviors will change, in addition to accepting the idea that there will be talk of a before and after COVID-19, which they considered to be an epochal event (*opportunities for change*, *a unique experience*, *being alone with yourself*) “*It is clear that an experience of this type will be remembered for life. I am 20 years old today, and what I often think about is how I will tell my children and grandchildren about this experience in the future, just like my grandparents told me about the war, its dangers and the fear that prevailed at that time.*”

The fourth and final code group, entitled “*self-evolution*,” focused on the positive aspects of temporal immobility, viewing everyday life from new perspectives and with new meanings. The suspension of everyday life activities allowed participants to find time to compare themselves with others, facilitating a process of self-reflection among some interviewees (*rediscovering oneself*, *achieving a new life balance*): “*I seek to*… *make this period a moment to become stronger and a chance to rediscover myself, as it is occurring in a completely new and undoubtedly singular context and situation.*”

This dramatic experience has caused many participants to develop a greater awareness of themselves and their own lives and to experience personal growth (*greater awareness*, *personal growth*) “*In the future we will overcome this crisis and return to normal, but in a more conscious way, knowing that we have acquired that freedom through our sacrifices.*”

### Continuity of Being

**FIGURE 1:**
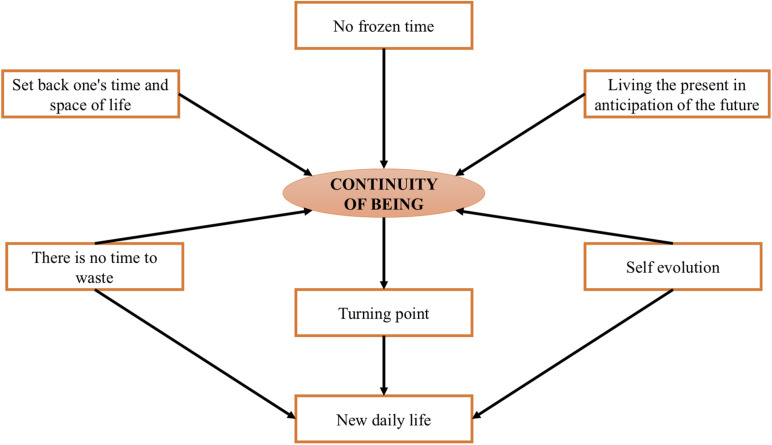
Core category, continuity of being.

Finally, the researchers created a core category labeled, “continuity of being” (see Figure 1).

In light of the proactive and empowering approaches to the lockdown adopted by many participants, we named the core category, “*continuity of being.*” This core category was characterized by maintaining or creating connections with past experiences, the present and striving toward one’s goals. In this regard, we considered the importance of beneficial past and future time perceptions during the lockdown as resilience strategies to cope with pandemic-related distress. The interviewees consistently highlighted the importance of maintaining time continuity and defining time and space during the lockdown as methods of turning this period of downtime into an opportunity for personal growth.

## Discussion

During the process of reading and interpreting the codes from this study and choosing participant quotations, the research team focused on the importance of the time-space divide that affected the interviewees’ emotions and thoughts. In fact, many interviewees reported changes in their daily time management in a confined space, in comparison with their usual activities. In the context of the COVID-19 pandemic, it would be interesting to understand any associations among people’s attitudes toward time and their levels of well-being, distress and anxiety. The experience of being spatially confined also confined many participants’ time perceptions to the present or in the past. However, for some participants, the lockdown has represented an unexpected opportunity to better understand themselves and to envision their futures.

Being confined in the present implied different meanings among the research participants. For multiple participants, being confined in the present has represented an opportunity to reorganize their time management and spaces, as well as to try new activities. However, for others, a present shaped by the lockdown represented a genuine obstacle that impeded their ability to envision new organizations of time and space, constructing their time perceptions of the present based upon their expectations to resume their pre-pandemic lives.

Conversely, other participants reported that home confinement entailed an excess of downtime that generated a state of stunned confusion, as well as perceptions of time characterized by feelings of suspension and immobility. Among these students, their lockdown experiences were framed by anguish, fear, uncertainty and sadness in relation to the recent events linked to the COVID-19 pandemic, in addition to an unrelenting focus on the current situation and an inability to shift their focus to past events or hope for the future “*There are many conflicting thoughts and feelings that crowd our minds these days, during which we find ourselves experiencing an uneasy and worrying situation. Sadness was definitely the emotion that primarily characterized the first days of the lockdown.*”

Furthermore, among participants, a common sense of loss emerged. Home confinement became a distressing experience in a context of heightened anxiety surrounding death. This suspended, uncertain time confronted the study participants with their most primitive fears, such as their fear of death as pandemic dreaming also highlighted (Iorio et al., 2020), while limiting ties and changes to their family structures.

Conversely, several interviewees reported feelings of confinement in the past, shaped by positive nostalgic memories of their past everyday lives prior to the COVID-19 lockdown (Zimbardo and Boyd, 2015). Additionally, among some young adults, the changes in their daily routines under lockdown served as an opportunity to open or deepen a dialogue with themselves, connecting with their pasts and envisioning a new future. A break from the unrelenting perceptions of time imposed by society fostered acceptance of the situation, in addition to the development of proactive attitudes. Envisioning the future implied opportunities for greater self-awareness and for participants to deeply reflect upon their lives and lifestyles.

Moreover, the lockdown experience resulted in freedom from pre-pandemic routines, as well as the need to cope with this new reality. Being trapped in the present while looking toward the future induced many young interviewees to rediscover the potentiality of space and to create new plans for their own futures. Therefore, confinement at home and spatial distance could be viewed as a sort of transitional experience or a potential turning point.

It is important to mention that, although many young respondents experienced negative feelings regarding the lockdown, some of them were able to view the resulting spatial and time restrictions in a positive light, transforming their time under lockdown into something productive and finding ‘*serenity in disorder.*’ As a result, interviewee’s experiences in a confined space led them to develop different time perceptions of the past, present and future, characterized by time subjectivations that seem to exclude the possibility for dialog among the three temporal perspectives.

Moreover, the core category, “*continuity of being*,” described a proactive experience, especially for young people, to overcome what Rosa (2003) defines as a type of de-temporalization that pushes people to think moment by moment. In addition, the storytelling design of the study facilitated the development of awareness surrounding subjective oppression and vulnerability, as well as common experiences of suffering, giving voice to people’s shared feelings, in line with community psychology perspectives.

Individual narratives encompass the expression of individual and collective experience-making processes. Narration involves biographical, reconstruction, reinsertion, recreation, realignment and relocation perspectives. Additionally, for some authors, narration also serves as a semiotic device mediating the connection between continuity and discontinuity, aimed not only at carrying out the previously mentioned functions, but also at creating new dynamic relations among them (Freda et al., 2015). Therefore, for participants, documenting their COVID-19 lockdown stories was an opportunity to reflect upon their lives that also acted as a resilience tool. In fact, opportunities mediated by creative expression, group discussions and shared actions aimed at individual and social awareness are a significant goal in community building, training and education (Carnevale et al., forthcoming; Arcidiacono et al., 2016; Di Napoli et al., 2019a). As a result, creating spaces in which people share their personal stories and ask questions about their common experiences is a preliminary goal of community psychology-based intervention strategies, while subsequently detecting meaning and symbolization in their interactions is the next step in promoting social awareness and community building (Arcidiacono, 2016; Procentese and Gatti, 2019; Procentese et al., 2019). Feeling part of a community where you share projects and consider the response of institutional members and referents reliable favors collective self-efficacy and the management of stress generated by this unforeseen emergency (Procentese et al., 2020).

### Limitations

Despite the contributions to the deepening of the existing literature, this study’s limitations included first of all its convenience sampling (non-probabilistic), so the generalization of results should be taken with caution. The unbalanced number of male and female respondents, although the sex composition of participants was representative of the general population of Italian psychology students (Arcidiacono and Tuozzi, 2017; Centro Studio Investimenti Sociali, 2019). Additionally, it is important to highlight the possibility that psychology students likely possess deeper reflective attitudes in comparison with students from other majors. Furthermore, the ethnic and geographical makeup of the sample was limited and was comprised solely of White university students living in a specific region of Italy. Therefore, any interpretation of the study results should be considered in conjunction with differing national and international contexts. Moreover, the researchers recommend that future studies also consider connections among young adults’ feelings regarding their physical and psychological well-being and their time perceptions. This study collected participants’ stories during the week with the highest number of reported COVID-19 cases in Italy; thus, it would be interesting to compare young peoples’ feelings and perceptions of their time under lockdown with their experiences during its de-escalation. The researchers further recommend that future studies explore any associations among time perceptions and well-being or negative emotions, in addition to comparing time perceptions across different phases of life.

## Conclusion

The space-time divide resulting from participants’ lockdown experiences was unexpected among the research team. Our students’ voices emphasized this aspect of the lockdown; thus, we sought to more profoundly explore this part of their experiences. Under lockdown, participants had to establish new time perceptions and new ways of “social sharing” to overcome physical confinement and distancing and to avoid further psychological distress in their daily lives.

According to Pichon-Rivière, in order to survive, hope must be planned through collective projects that help people face difficulties and changes. Planning hope emerges from people’s abilities to create alternatives to collectively share spaces, such as singing from balconies in the context of the lockdown, as described by Di Napoli et al. (2021). The implications and impacts of the research findings could help in preparing plans and providing services in the context of a generalized pandemic. In this regard, the study findings help us recognize the impact of limits on one’s actions and/or mobility on individuals’ psyches. The young adult study participants described their lockdown experiences through varying perceptions of time, with some of them reliving their pasts, while others focused on rethinking and shaping their futures. The COVID-19 pandemic altered participants’ lives in a spatial sense, relegating them to spend the days under lockdown reliving past connections, renewing contact with old friends and lovers and redefining their future expectations.

Moroccan anthropologist Zakaria Rhani (2019) documented experiences of severe confinement in Morocco during the Years of Lead (1956–1999). His interview with a political prisoner who spent most of his life confined in Tazmamart, a cold and secluded prison in the Atlas Mountains, best characterizes some of the effects of extreme confinement. In order to survive confinement in such a desolate space, the prisoner, Kawni, described how his body lived confined in prison, suffering from cold and hunger, while allowing his soul the opportunity to continue life far away from these deplorable conditions. Therefore, this body-soul divide allows individuals’ spirits to survive in the face of repression and limits to individual freedoms. Reflecting upon this extreme situation aids in understanding how individual experiences of forced confinement may result in different time and space perceptions. In this regard, it is important to reflect upon how the effects of confinement on people’s everyday lives results in the development of a wide array of individual resilience strategies.

The lockdown period has deprived people of their health, families and careers, but also of small everyday things, like grabbing a coffee in the morning or with friends, a kiss or a hug, affection, feelings, nature and the power of sharing. Furthermore, home confinement under lockdown implied an absence of close physical contact and the presence of other people in our lives. Therefore, maintaining continuity with past experiences and future goals, without becoming trapped in the present, is a crucial need and a psychological resilience strategy to maintain life continuity. Tools and spaces for sharing during emergencies, became a goal. It will help to lower distress and favor a recovery path of continuity with the past and with new future perspectives. The continuity of time and space to meet and interact is a basic need that characterizes young people’s life contributing to their well-being (Di Napoli et al., 2019b). Therefore, the core category of this research “continuity of being” emphasizes the role of time and moreover of shared time allowing the individual and relational experience of connectedness.

The soundness of these findings suggests the importance to take into account the perception of time, especially among young people that particularly suffer for their space limitation and lack of social interactions. Moreover, it would be interesting to devote some studies to the effect of lockdown space limitation on children with dyslexia already affected by time processing difficulties (Casini et al., 2017). In such cases tailor specific supportive interventions for their families may help in managing time/space limits of forced home confinement.

In the words of Ernest Pichon-Rivière (1985), from our youth we learned that in times of uncertainty and a “lack of hope,” it is essential to undertake collective projects and to plan shared hope. Thinking on youth experiences in pandemic the public bodies have to be able to sustain with actions and projects their need of connectedness with their own experiences and the world around them.

## Data Availability Statement

The raw data supporting the conclusions of this article will be made available by the authors, without undue reservation.

## Ethics Statement

Studies involving human participants were reviewed and approved by the University of Naples Federico II Department of Humanities’ Ethics Board for Research in Psychology (March 15, 2020). Participants provided written informed consent to participate in this study. Written informed consent was obtained from the individual(s) for the publication of any potentially identifiable images or data included in this article.

## Author Contributions

All authors listed have made substantial, direct and intellectual contributions to this research, and approved it for publication.

## Conflict of Interest

The authors declare that the research was conducted in the absence of any commercial or financial relationships that could be construed as a potential conflict of interest.
